# Sedation and general anaesthesia of crocodilians: a systematic review

**DOI:** 10.1186/s13028-024-00779-1

**Published:** 2024-10-24

**Authors:** Asbjørn Onsberg Kruuse, Louise Damholt Markusen, Carsten Grøndahl, Lisbeth Høier Olsen

**Affiliations:** 1 Department of Veterinary and Animal Sciences, Faculty of Health and Medical Sciences, University of Copenhagen, Ridebanevej 9, 1870 Frederiksberg C, Denmark; 2 Centre for Zoo and Wild Animal Health, Copenhagen Zoo, Frederiksberg C, Denmark

**Keywords:** Alfaxalone, *Alligator mississippiensis*, Alpha-2-adrenoceptor agonists, *Crocodylus johnstoni*, *Crocodylus niloticus*, *Crocodylus porosus*, Dissociative anaesthetics, Inhalation anaesthesia, Ketamine, Righting reflex

## Abstract

Sedation and general anaesthesia of crocodilians pose unique challenges due to their aggressive nature, poikilothermic physiology, and specific anatomical and physiological characteristics, all factors that complicate crocodilian anaesthesia. This review aimed to systematically review the literature regarding sedation and general anaesthesia of crocodilians with focus on efficacy and impact on vital parameters. A systematic literature search was performed according to PRISMA guidelines on May 2, 2023 in the databases Embase, PubMed, Scopus and Web of Science. Publications were excluded based on predefined exclusion criteria, which encompassed non-standard publications and publications unrelated to crocodilians, with fewer than five animals and/or with insufficient data on sedation and general anaesthesia. Five key factors were used to evaluate the strength of evidence: number of included animals, study design, definition of recovery time, blinded assessment of recovery and conflict of interest. Ten publications were included in this systematic review. Drugs used included alpha-2-adrenoceptor agonists, dissociative anaesthetics, benzodiazepines, neuromuscular blocking agents, propofol, alfaxalone, and inhalant gasses. The studies included in total 55 *Alligator mississippiensis*, 110 *Crocodylus porosus*, 15 *Crocodylus johnstoni*, and 15 *Crocodylus niloticus*. Factors such as temperature, administration route, dose, species, and age influenced protocols for sedation and general anaesthesia of crocodilians. The studies included used five different study designs. Only one study included a control group, done on retrospectively collected data. Blinded recovery assessments and declarations of no conflict of interest were noted in some studies. The use of four distinct recovery definitions posed challenges to comparability in this systematic review. The studies reported that medetomidine provided stable and reversible sedation, although it depressed heart rate. Alfaxalone was less stable outside the optimal temperature range. Intubation and inhalation anaesthesia were effective, and adrenaline reduced the length of the recovery period. Overall, the review provides valuable insights for veterinarians, researchers, and wildlife professionals involved in sedation and general anaesthesia of the crocodilian species, however, the literature is limited, and further research is needed to improve evidence-based medical management.

## Background

Crocodilians are intimidating creatures capable of inflicting serious damage to personnel, themselves, and their surroundings. Therefore, the number one priority should be safety. The physical restraint of crocodilians is a dangerous undertaking, and handling-associated stress can result in altered behaviours and metabolic changes [1]. In light of that, sedation and general anaesthesia can pose a safer alternative that alleviates stress to acceptable levels. Previous methods relied on neuromuscular blocking agents such as gallamine, atracurium, and pancuronium as the sole immobilising agent [2]. This however poses welfare concerns as they are neither analgetic, tranquilizing, sedative nor anaesthetic [2]. Today, neuromuscular blocking agents are still in use but are normally not used without local anaesthetics, sedatives and/or general anaesthetics depending on the procedure [2–4].

Crocodilians have unique anatomical and physiological traits including cardiovascular abilities such as blood shunting from the left to the right aortic arch through the foramen of Panizza, constriction of the pulmonary arteries via the cog-teeth-like valve and a renal portal shunting mechanism [5, 6]. Additionally, crocodilians are poikilothermic animals with a preferred optimal thermal zone (POTZ) of 30–32 ℃ [1, 7, 8]. These distinctive anatomical structures and physiological functions play an important role in the crocodilian’s semiaquatic life, but, simultaneously, they seem to create difficulties during general anaesthesia [2, 5, 9].

Sedation and anaesthesia of crocodilians are not only relevant for safety, immobilisation, and translocation but are also crucial aspects of medical care, such as diagnostics, treatments, and surgical procedures. The objective was to systematically review the literature regarding sedation and general anaesthesia in crocodilians with focus on the efficacy and impact on vital parameters. Monitoring depth of anaesthesia, respiratory parameters, and circulatory status, and the importance of factors such as temperature, administration route, age, and species will be addressed. Finally, specific physiological and anatomical factors that present hurdles in achieving optimal sedation, immobilisation and general anaesthesia will be discussed.

### Search strategy

This systematic review was performed using the Preferred Reporting Items for Systematic Reviews and Meta-Analyses (PRISMA) guidelines [10]. The search string was constructed using the PICO method (Population/People/Problem, Interventions, Comparison/Control, Outcome) [11]. The population was defined as members of the Crocodilia order [2]. However, galvianae and tomistominae were excluded due to preliminary searches indicating a lack of publications regarding these species, leaving only crocodiles, alligators, and caimans relevant for the final search string. The intervention was defined as sedation and general anaesthesia. There were no requirements for comparison/control since preliminary searches showed very few publications with control groups. Lastly, the recorded outcomes were defined as monitored vital parameters (heart rate (HR), respiratory rate (RR) and temperature) and/or efficacy (level of sedation and general anaesthesia). These terms were however not included in the search string to avoid leaving out relevant literature. The above considerations led to the following search string:

(crocodil* OR crocodylia OR alligator? OR caiman?) AND (anesthesia OR anaesthesia OR sedation OR immobilization OR immobilisation OR analgesia).

“Analgesia” was included as a search item, but publications addressing analgesia were later excluded in order to improve the focus of the review. The definitive search was conducted on the 2nd of May 2023, in the databases Embase, PubMed, Web of Science, and Scopus. The retrieved publications were imported into EndNote from the search databases and automation tools were used to identify duplicates, nonstandard publications, and publications not written in Danish or English. Then manual screening was performed to identify publications for exclusion that automation tools did not catch. The screening was performed in two rounds: title and abstract screening and thereafter full-text screening. Publications that met any of the exclusion criteria below, were excluded from the present review:

- Publications that do not relate to members of the Crocodilia order.
- Publications that do not have sedation, immobilisation, or general anaesthesia as the main topic.
- Publications that are not standard papers (case studies, review articles, conference abstracts, editorials, notes, and letters).
- Publications that do not include information on anaesthesia depth and monitoring of vital parameters and/or levels of sedation or general anaesthesia.
- Publications that include fewer than five animals.

In order to reduce bias during the literature search, the selection of databases and exclusion criteria were defined before the search and all screening and exclusion were performed by two authors (AOK and LDM). Re-screening and consensus were obtained in case of disagreement.

To evaluate bias in the included publications, the strength of evidence (SOE) in each publication was addressed with a focus on study design, number of included animals, presence of blinded assessment of recovery, reported conflict of interest and definition of recovery.

## Review

### Results

**Fig. 1:**
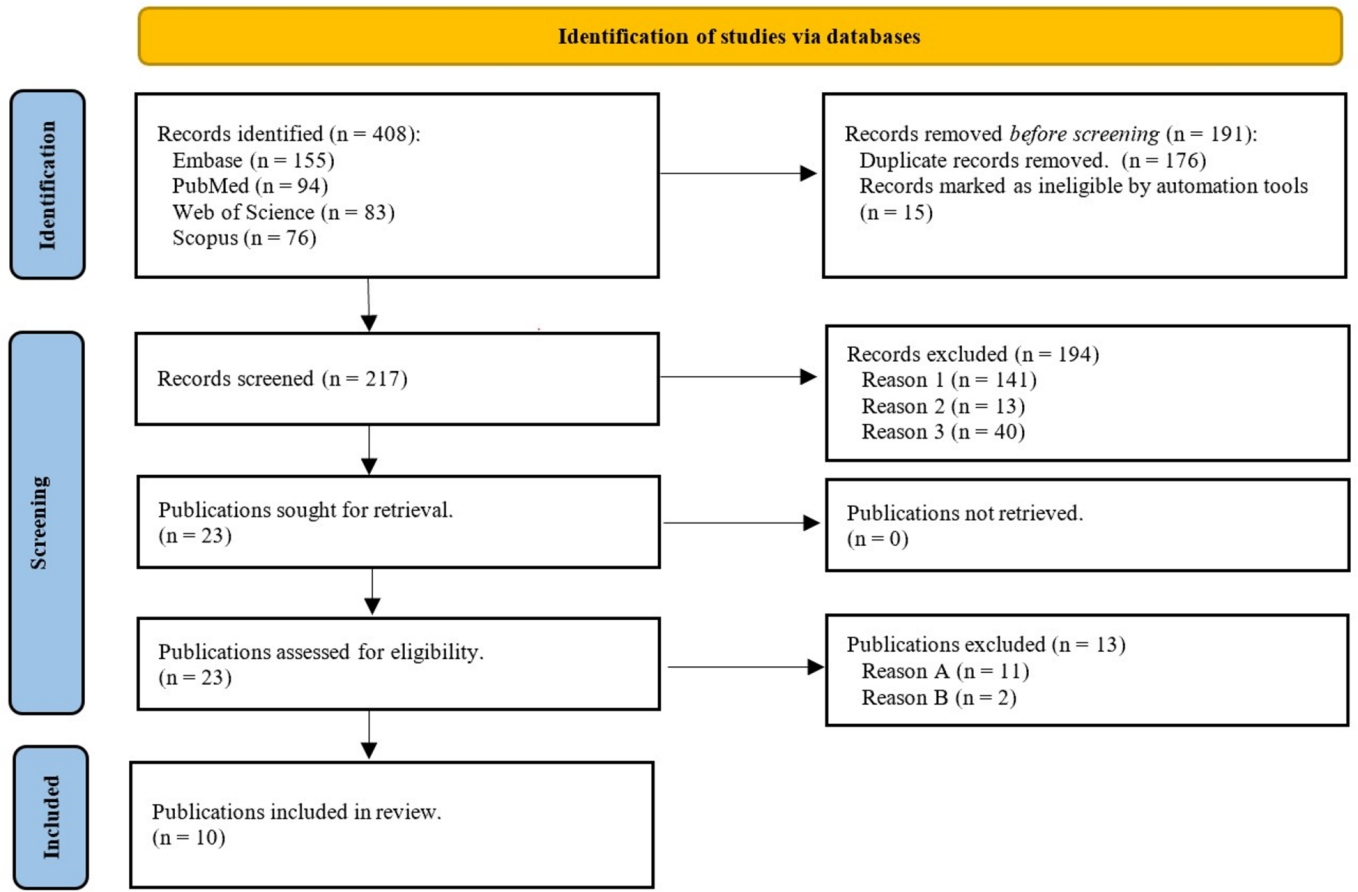
Flowchart illustrating the review process. Number of publications incorporated from search databases and number of excluded publications at each corresponding step are illustrated. Reason 1: Records that do not relate to members of the Crocodilia order. Reason 2: Records that are not standard papers. Reason 3: Records that do not have anaesthesia, sedation, or medical immobilisation as the main topic. Reason A: Publications that do not include documentation of depth of anaesthesia and measurement of vital parameters. Reason B: Publications that include less than five animals

Number of excluded and included publications are summarised and visualised in Fig. 1 according to PRISMA [10] guidelines. In brief, a total of 408 publications resulted from searching four databases. The following screening process excluded 398 publications based on duplicates, language, and the abovementioned exclusion criteria. The remaining ten publications were included in the review.

#### Origins and human habituation

The ten publications encompass a range of settings and origins each presenting unique scenarios with distinct requirements for anaesthesia protocols. An overview of the origins of animals and whether they were human-habituated or not are provided in Table 1. None of the included publications were field studies and the studies were either conducted on-site (farm or zoo) or at a nearby veterinary facility.

**Table 1:** An overview of the animal’s origin and human habituation

	References									
	[1]	[3]	[4]	[6]	[7]	[8]	[9]	[12]	[13]	[14]
Origin	Farm	Wild caught	Zoo	Farm	Farm	Farm	Zoo	Farm	Captive bred	Juveniles: Captive bred Adults: Wild caught
Human habituated	–*	No	–	No	–*	No	–	Yes	Yes	Juveniles: – Adults: No

– = Missing data or no information. *Although no information is provided on human habituation of these animals, the animals are from the same venue as reference nr. 6 and 14 where the animals were not habituated to humans

The publications include data on 55 alligators (*Alligator mississippiensis*), 110 saltwater crocodiles (*Crocodylus porosus*), 15 freshwater crocodiles (*Crocodylus johnstoni*), and 15 Nile crocodiles (*Crocodylus niloticus*). Factors affecting SOE are summarised in Table 2.

**Table 2:** Overview of the ten included publications and factors evaluated

Factors affecting strength of evidence					
Reference	Study design	No. of animals	Blinded assessment of recovery	Conflict of interest	Definition of recovery
[3]	PCS	8	–	–	A
[6]	PRS	40	Yes	–	C
[1]	PRS	20	–	–	D
[7]	PRS	40	Yes	–	C
[8]	PRSX	25	Yes	–	D
[9]	PRSX	5	Yes	–	A/B
[4]	PS	26	–	–	A
[12]	PS	5	–	No conflict of interest declared	D
[13]	PS	10	–	No conflict of interest declared	B
[14]	PSX	16	–	–	B

*PCS* prospective controlled study, *PRS* prospective randomised study, *PRSX* prospective randomized study with crossover, *PS* prospective study, *PSX* prospective study with crossover, *A* based on the return of reflexes from induction, *B* based on the return of reflexes from the administration of the reversal agent, *C* based on the return of all reflexes and the ability to walk away from the administration of reversal agent, *D* duration of immobilisation from induction,—missing data/no information

The anaesthesia protocols used are listed in Tables 3 and 4.

**Table 3:** An overview of the different anaesthetic protocols used  for *Crocodylus* species

Protocols used for *Crocodylus* species							
Drug combination administration							
(Dose [mg/kg])	Route	Species	Body weight [kg]	Induction time [min]	Recovery time [min]		Refs.
Medetomidine (0.5)	IM TL	*C. porosus*	2.0–4.8	15–30 $\pm$ 10 ^(1)^	10 $\pm \,5$ ^(2)^		[7]
	IM TL	*C. porosus*	3.6–11.3	30	120–135		[6]
	IM TL	*C. porosus*	5.6–8.1	30	15–30 ^(2)^		
	IM Tail	*C. porosus*	3.7–7.3	Only sedation			
	IM PL	*C. porosus*	3.0–8.1	Only sedation			
	IM TL	*C. johnstoni*	8.5–12.2	Not immobilised	90		
Medetomidine (0.75)	IM TL	*C. johnstoni*	4.1–7.6	30–45	90		
Medetomidine (0.5–2)	IM TL	*C. porosus*	1–2	Not immobilised	15 ^(2)^		
Medetomidine (0.25–1)	IV OS	*C. porosus*	1–2	Not immobilised	_		
Medetomidine (0.3) Ketamine (15) Sevoflurane †	IM TL	*C. niloticus*	4.2 $\pm$ 1.7	16 $\pm$ 8	20–60		[13]
Medetomidine (0.0063–0.0106) Propofol (1.6–3.8) Isoflurane †	IM TL IV OS	*C. niloticus*	21–47	_	123–195^(3)^		[12]
Midazolam (5)	IM TL	*C. porosus*	2–3.5	15	60		[1]
Alfaxalone	IV OS	*C. porosus*	0.6–2.5	5	10–105^(1)^		[8]
(3)			0.2–0.6	5	10–55^(1)^		

Protocols are for *C. porosus* = *Crocodylus porosus* (saltwater crocodile)*, C. johnstoni* = *Crocodylus johnstoni* (freshwater crocodile)*, C. niloticus* = *Crocodylus niloticus* (Nile crocodile). Drugs administered at the same time, unless else is indicated. The protocols used different routes of administration

^1^Depends on body temperature

^2^Reversed with atipamezole

^3^Duration of inhalation anaesthesia. †Inhalation anaesthesia after immobilisation and intubation.—= missing data or no information

**Table 4:** Overview of the different anaesthetic protocols used in the included publications for *Alligator mississippiensis* (Alligator)

Protocols used for *Alligator mississippiensis*							
Drug combination							
Drug 1 (Dose [mg/kg])	Drug 2 (Dose [mg/kg])	Administration route	Body weight [kg]	Induction time [min]	Recovery time [min]		Refs.
Medetomidine (0.22 $\pm 0.$ 076)	Ketamine (10.0 $\pm$ 4.9)	IM masseter or TL	6.75 $\pm$ 1.02	20 $\pm 9$	35 $\pm$ 22 ^(3)^		[14]
Medetomidine (0.13 $\pm 0.019$)	Ketamine (7.5 $\pm$ 4.2)	IM masseter or TL	36.7 $\pm$ 38.9	27 $\pm$ 17	38 $\pm$ 20 ^(3)^		
Diazepam (0.22–0.62)	Succinylcholine (0.14–0.37)	IM PL	40–106	5–15 ^(4)^	–		[4]
Diazepam (0.4)	Atracurium (4) ^(6)^	IM TL	1.5–3.3	15–90	270–360		[3]
Tiletamine ^(5)^ (7.5)	Zolazepam ^(5)^ (7.5)	IM TL	1.5–3.3	53 $\pm$ 31^(1)^	183 $\pm$ 34		
Isoflurane 5%	Isoflurane^(7)^ 3%	Inhalation	6.3 $\pm$ 1.4		51 $\pm$ 16^(2)^ (adrenaline) 107 $\pm$ 44^(2)^ (saline)		[9]

Drugs administered at the same time, unless else is indicated

*IM* intramuscular, *TL* thoracic limb, *PL* pelvic limb

^1^ Time to peak effect defined as slowest righting time (none of these animals lost the righting reflex)

^2^ Time to extubating

^3^ Reversed with atipamezole

^4^ After succinylcholine chloride

^5^ Telazol at 15 mg/kg

^6^ Given 15 min after diazepam

^7^ After loss of spontaneous movement and response to cloacal stimulation.—= missing data or no information

#### Choice of drugs

As shown in Tables 3 and 4, medetomidine was the most frequently used sedative [6, 7, 12–14]. Ketamine [13, 14] and diazepam [3, 4] were also used, although only in combination with other drugs.

The included publications reported a wide range of dosages when using medetomidine. Generally, higher dosages were used, when medetomidine was used alone, whereas lower dosages were used when combined with other drugs [12–14]. Recovery time during medetomidine sedation was dependent on whether atipamezole was administered as an antidote at the end of the procedure (see Tables 3 and 4) [6, 7, 12–14].

It is important to note that in one study using medetomidine without atipamezole as an antagonist, distension of the coelom and a risk of re-sedation were found if the animals were left unstimulated [6].

The combination of medetomidine and ketamine was reported to provide reliable, safe, and reversible general anaesthesia without severe cardiac and respiratory depression [7, 14]. One study used a combination of intramuscular (IM) medetomidine and intravenous (IV) propofol before inhalation anaesthesia with isoflurane in a circle rebreathing circuit. Isoflurane was set to 4% with a flow rate of 2 L/min and manual positive pressure ventilation was applied [12].

Two of the included publications used diazepam as the initial tranquillizer [3, 4]. Both studies used diazepam in combination with a neuromuscular blocking agent (NMBA). One of these studies combined diazepam with succinylcholine IM and found that the combination provided reduced awareness and efficient immobilisation while also allowing for a lower dose of succinylcholine [4]. The other study combined diazepam with atracurium IM and found that atracurium induced prolonged apnoea [3]. The same study had another group of animals immobilised by a combination of tiletamine and zolazepam IM. Induction and recovery times (see Table 4) were longer compared to studies using medetomidine however no effect on vital signs was reported [3].

A study compared changes in vital parameters and circulating biomarkers between two groups of crocodiles. One group was tranquilised with midazolam and another group was exposed to physical restraint [1]. Crocodiles tranquillized with midazolam had minimal changes in vital parameters and circulating biomarkers (lactate, blood pH and carbon dioxide (CO_2_)) compared to the group of crocodiles exposed to physical restraint [1].

Another study used alfaxalone IV as the sole anaesthetic agent [8]. Although induction was consistent using alfaxalone at different temperatures, the study revealed a significant decrease in HR, sudden bursts of aggression, hypersensitivity, muscle twitching, and uncontrolled, involuntary movements of the limbs which led the researchers to conclude that alfaxalone is a temperature-dependent and unpredictable immobiliser. In some cases, prolonged apnoea was observed even when the patient was no longer immobilised [8].

One study used only isoflurane to immobilise crocodilians. The animals (body weight range 4.6–8.0 kg) were intubated while physically restrained and thereafter connected to a semi-closed rebreathing circuit. General anaesthesia was induced using 5% isoflurane in 100% oxygen at a flow rate of 1 L/min [9]. Once spontaneous movement was lost, and alligators did not respond to cloacal stimulation, isoflurane was decreased to 3%. A variable recovery time was reported, however, usage of 0.1 mg/kg adrenaline IM was found to significantly reduce the time to return of spontaneous movement and extubation [9].

The most recent study used a combination of medetomidine and ketamine IM before intubation and connection to a circle system to deliver sevoflurane inhalation anaesthesia in oxygen with pressure-controlled mechanical ventilation [13]. Atipamezole IM was used to antagonise medetomidine at the end of surgery. No perioperative complications were reported using this protocol [13].

#### Monitoring depth of sedation and general anaesthesia and vital parameters

The depth of sedation and general anaesthesia was monitored using different reflexes and responses (Table 5).

**Table 5:** Number of publications (out of ten) in which a given reflex was observed and reported

	Reflexes or responses					
	Righting reflex	Pinch withdrawal reflex	Palpebral reflex	Corneal reflex	Palatal valve	Other ^(1)^
Number of publications	8	8	7	3	3	7

^1^ Other reflexes or responses including digital pressure applied to flanks (n = 2), biting reflex (n = 2), ocular response (n = 1), cloacal palpation (n = 1), position of eyelid (n = 1)

In one study the tail and toe pinch withdrawal reflex (PWR) were compared [13]. The tail PWR was reported as a more useful indicator compared to the toe PWR. The righting reflex was also assessed, but they found it unreliable as the crocodiles could keep their heads elevated and respond to noxious stimuli without turning into sternal recumbency [13].

In terms of monitoring vital parameters, all publications included observations on HR and the majority of the publications reported RR as well. Temperatures were recorded as either body temperature [6, 7, 9, 12–14] or ambient temperature [1, 3, 4, 8]. Some studies recorded heart rate using electrocardiography, ultrasonography [4, 14] or blood pressure [6, 12] (Tables 6 and 7).

**Table 6:** Monitoring of vital parameters

Intervention	Heart rate [beats/min] Respiratory rate [breaths/min]				Refs.
	Before sedation/general anaesthesia	During sedation/general anaesthesia	Reported cardiorespiratory complications		
Diazepam (0.37 mg/kg IM) Succinylcholine (0.24 mg/kg IM)	_	HR: 44 (8–60) RR: 13 (1–32)	No observed		[4]
Diazepam (0.4 mg/kg IM) Atracurium (4 mg/kg IM)	HR: 58 ± 8 RR: 11	HR: No change RR: –	Apnea		[3]
Tiletamine-zolazepam (15 mg/kg IM)	HR: 56 ± 3 RR: 12 ± 8	No change			
Medetomidine (220.1 ± 76.0 µg/kg IM) Ketamine (10.0 ± 4.9 mg/kg IM)	HR: 37 ± 4 RR: 8 ± 2	HR: 13 ± 5 RR: 6 ± 4	No observed		[14]
Medetomidine (131.1 ± 19.5 µg/kg IM) Ketamine (7.5 ± 4.2 mg/kg IM)	HR: 24 ± 45 RR: 8 ± 2	HR: 22 ± 6 RR: 6 ± 2	Cardiac arrythmias in 2 animals ^(1)^		
Medetomidine (300 µg/kg IM) Ketamine (15 mg/kg IM)	HR: 50 ± 10 RR: 10 ± 6	HR: Significantly decreased	Bradycardia		[13]
Medetomidine (500 µg/kg IM)	HR: 51 ± 7	HR: Significantly decreased			[6]
Medetomidine (250–2000 µg/kg) IM or IV	IM group HR: 66 ± 5 IV group HR: 71 ± 4	IM group HR: 40 ± 12 IV group HR: 43 ± 11	Bradycardia in 2 animals		
Medetomidine (500 µg/kg IM)	_	_			
Medetomidine (750 µg/kg IM)	_	_	Bradycardia		
Medetomidine (500 µg/kg IM)	†	†	Bradycardia		[7]
Midazolam (5 mg/kg IM)	†	†	Bradycardia		[1]
Alfaxalone (3 mg/kg IV)	HR: 32–80 ^(1)^	HR: 26–68 ^(2)^	Apnea		[8]
5% induction, 3% maintenance Flow rate of 1L/min	_	_	No observed complications		[9]
Medetomidine (8.3 µg/kg IM) ^(3)^ Propofol (2.1 mg/kg IV)	_	HR: 21	No observed complications		[12]

During sedation and general anaesthesia of crocodilians.—= not applicable. † = Only shown in a figure in the publication. Heart rate before anaesthesia varied within the given range

^1^Second-degree atrioventricular block and premature ventricular complexes (not considered life-threatening)

^2^Heart rate during anaesthesia decreased more at lower thermal zones and varied within the given range

^3^Calculated average

**Table 7:** Intramuscular immobilisation at four different thermal zones

			Time to immobilisation at given temperature [min]				Length of anaesthesia at given temperature [min]				Recovery from time of first injection [min]			
Animals	Drug combination (dose [mg/kg])	Route	17–21 °C	22–26 °C	27–29 °C	30–32 °C	17–21 °C	22–26 °C	27–29 °C	30–32 °C	Reversal agent (dose[mg/kg])	Time [min]		References
*A. mississi-ppiensis* (n = 7)	Medetomidine (0.22) Ketamine (10.0)	IM TL		20 ± 9				112 ± 45			Atipamezole (1.18)	35 ± 22		[14]
Adult *A. mississi-ppiensis* (n = 9)	Medetomidine (0.13) Ketamine (7.5)	IM TL		27 ± 17				161 ± 35			Atipamezole (0.694)	38 ± 20		
*C. niloticus* (n = 8)	Medetomidine (0.3) Ketamine (15)	IM TL			16 ± 8				MA		Atipamezole (1–3)	36 ± 12		[13]
*C. porosus* (n = 10) ^(2)^	Medetomidine (0.5)	IM TL			30				98 ^(1)^		No reversal (n = 5)	120–135 (Post first injection)		[6]
											Atipamezole (n = 5) (2.5)	15 ± 6		
*C. johnstoni* (n = 5)	Medetomidine (0.75)	IM TL			30 ± 15				60 ^(1)^		No reversal	90 (Post first injection)		
*C. porosus* (n = 40) ^(3)^	Medetomidine (0.5)	IM TL	NI	35 ± 15	35 ± 10	15 ± 10	RV50	RV50	RV50	RV50	Atipamezole (2.5)	10 ± 5		[7]
*A. mississi-ppiensis* (n = 8)	Tiletamine-Zolazepam (15)	IM TL				53 ± 31				131 ^(1)^	No reversal	183 ± 34 (Post first injection)		[3]
*A. mississi-ppiensis* (n = 8)	Diazepam (0.4) 15 min Atracurium (4)	IM TL			39 ± 34						Neostigmine No reversal ^(4)^	317 ± 36 (Post first injection)		
Adult *A. mississi-ppiensis* (n = 28)	Diazepam (0.37) 20 min Succinylcholine (0.24)	IM HL		25–35							No reversal	180 +		[4]
*C. porosus* (n = 10)	Midazolam (5)	IM TL				15					No reversal	60		[1]

All animals were juvenile unless else is indicated. *A. mississippiensis* = *Alligator mississippiensis* (Alligator), *C. porosus* = *Crocodylus porosus* (saltwater crocodile), *C. johnstoni* = *Crocodylus johnstoni* (freshwater crocodile), *C. niloticus* = *Crocodylus niloticus* (Nile crocodile)

*TL* Thoracic limb, *HL* Hind limb, *NI* Not immobilised, *RV50* reversed with atipamezole after 50 min

MA = Maintained with inhalation anaesthesia and is therefore not applicable

^1^Calculated value, recovery time—time to immobilisation

^2^Animals injected in the pelvic limb and tail are not included in this Table

^3^Divided into four groups which were sedated at four different temperatures

^4^Three animals were attempted reversed with three different doses of neostigmine with no effect and reversal agents were not administered to the remainder of the animals

#### Heart rate and respiratory rate

The protocol including diazepam (0.37 mg/kg) followed by succinylcholine chloride (0.24 mg/kg) had limited effect on respiratory and cardiovascular parameters [4]. Medetomidine (220 ± 76 µg/kg) combined with ketamine (10.0 ± 4.9 mg/kg), however, significantly reduced heart rate (HR) (38–65%) and respiratory rate (RR) (27–40%) compared to baseline in juvenile animals (body weight range 4.96–8.0 kg) [14]. In the same study, an adult group of animals (body weight range 14.0–154.0 kg) was anaesthetised using the same combination but at a lower dose [14]. Here, the HR also decreased (26.9–33.9% compared to baseline) and two animals experienced few cardiac arrhythmias (second-degree atrioventricular block and premature ventricular contractions), however the arrhythmias were not considered life-threatening and were resolved after administration of atipamezole. In other included studies no significant changes in HR were reported using isoflurane starting at 5% induction and reducing to 3% maintenance [9], tiletamine-zolazepam (15 mg/kg) [3] or a combination of diazepam (0.4 mg/kg) and atracurium (4 mg/kg) [3]. Two studies did however observe respiratory complications such as bradypnea or apnoea [3, 8].

When medetomidine was used alone, a number of publications reported varying degrees of effect on the cardiovascular and respiratory systems. One study compared different doses of medetomidine and reported a significant reduction in HR during sedation [7]. The freshwater crocodiles’ group in the study needed a higher dose of medetomidine at 750 µg/kg and was not reversed with atipamezole, resulting in complications the next day including re-sedation, distention of coelom, bradycardia, and slow responses [7].

#### Temperature

In the ten included publications, crocodilians were sedated and anaesthetised in different thermal zones. The thermal zone 30–32 ℃ represents the crocodilian’s preferred optimal thermal zone (POTZ) [1, 7, 8].

A study [8] reported that alfaxalone was unreliable both at POTZ and at temperatures below POTZ. They noted that the further from POTZ, the more animals would drift in and out of immobilisation with a tendency to re-immobilise if left without stimulation. In a later study, medetomidine was tested at 500 µg/kg IM in the thoracic limb (TL) and a much more reliable immobilisation was observed at sub-optimal thermal zones [7]. Medetomidine provided safe and reversible immobilisation, with no incidents of apnoea and no need to intubate the animals. They also concluded that temperature has a consistent effect on the pre-anaesthetic HR by decreasing baseline values. Administering medetomidine would initially cause bradycardia after which the HR would stabilise, regardless of whether immobilisation had been achieved. Even though medetomidine performed better at sub-optimal thermal zones, there was a significant difference between induction times with crocodilians only being sedated and not anaesthetised at 17 ℃ [7].

#### Age, size, and species

A study immobilised 30 saltwater crocodiles by administering medetomidine IM or IV [7]. The age distribution was primarily juvenile crocodiles with a weight group of 1–2 kg and a weight group of 3–11.3 kg. They were unable to immobilise crocodiles weighing between 1–2 kg even though they received up to 4 times the dose used in the heavier group and up to 1 mg/kg IV, whereas the older group (3–11.3 kg) was immobilised with only 500 µg/kg IM in the TL. A difference in doses needed to immobilise crocodilians in different weight groups was also noted by another study [14], who anaesthetised a group of juvenile alligators (6.75 ± 1.02 kg) and a group of adult alligators (36.65 ± 38.85 kg). In this study, the juvenile animals needed a significantly higher dose of medetomidine and tended to require a higher dose of ketamine, than needed in the adult animals for the same outcome [14].

It was also found that freshwater crocodiles needed 50% higher doses for the same outcome produced in saltwater crocodiles [7]. Furthermore, the study showed that administrating 500 µg/kg medetomidine IM in the pelvic limb or the tail, made a significant difference from the administration of the same dose in the TL, where no immobilisation and only light sedation occurred when administered in the caudal body part [7]. Looking at the rest of the publications included in the present review, only one other publication used the pelvic limb as an administration route [4]. In this publication, diazepam and succinylcholine were administered with no complications or need for re-dosing.

## Discussion

The results can be divided into two general outcomes: efficacy of sedation and general anaesthesia and in addition, monitoring of vital parameters. Among the ten included studies, medetomidine appeared to be the most versatile drug as it led to reliable, safe, and reversible chemical immobilisation [6, 7, 15] in multiple temperature zones [7]. Combining medetomidine with a dissociative agent such as ketamine seemed to further increase the reliability of the chemical immobilisation [13, 14]. Alfaxalone on its own administered IV appeared however only useable under controlled environments in the POTZ with intubation and ventilation available [8]. In the following, physiological challenges regarding crocodilian anaesthesia will be discussed and different anaesthetic protocols will be compared with a focus on vital parameters, recovery, and complications.

### Physiological challenges

The heterogeneity of outcomes during sedation and general anaesthesia might be related to the unique physiology of crocodilians. Crocodilians have a functionally and hemodynamically sophisticated cardiovascular system, where shunting and mixing of oxygenated and nonoxygenated blood is a physiologically controlled mechanism, enabling long periods of submersion and apnoea [5]. When submerged, pulmonary hypertension and constriction of the cog-teeth-like valve shunt blood from the right ventricle into the left aorta and systemic circulation thereby shunting blood away from the lungs [16]. This can lead to prolonged recovery times following inhalation anaesthesia in crocodilians, due to decreased exhalation of the anaesthetic agent [8, 17].

A study [18] anaesthetised the reptiles Dumeril’s monitors (*Varanus dumerili*) and reported recovery times of 71 min (± 16 min) using 5% isoflurane in 100% oxygen. Using the same protocol another study recorded longer recovery times (98 min (± 42 min)) in alligators [9]. It can be speculated that the difference in recovery time between the two studies is associated with species differences in the cardiovascular system of crocodilians and other reptiles. Possible species differences in minimal alveolar concentration (MAC) could also have influenced the findings [19], however to the authors’ knowledge, no studies have reported MAC values for reptiles belonging to the order of Crocodilia. To expedite the recovery process in crocodilians, adrenaline was administered IM, which resulted in a significant reduction in recovery time [9]. They speculated on the potential effect of adrenaline on the cog-teeth-like valve and the foramen of Panizza, the anatomical structures involved in shunting blood away from the lungs in crocodilians. These findings highlight the importance of interventions that can accelerate recovery and minimise the physiological stress associated with prolonged anaesthesia in crocodilians, providing an interesting topic for further research.

The renal portal system (RPS) is present in most reptiles and certain bird species. The pharmacological importance of the RPS is subject to debate in the literature. A study [20] reported that the RPS in the common green iguana (*Iguana iguana*) only had clinical relevance when administering drugs with a high first-pass tubular excretory rate such as penicillin. Additionally, diazepam is metabolised into active metabolites in the liver of humans, rats, and dogs [21] and succinylcholine is metabolised by plasma cholinesterase in other species of reptiles [22] thereby offering an explanation as to why a study [3] could administer diazepam and succinylcholine in the caudal vein without a first pass effect. The RPS was suggested as an explanation for why a study [6], was unable to immobilise crocodilians with medetomidine injection in caudal sites, suggesting that medetomidine had undergone a first-pass effect, significantly lowering systemic concentrations. Interesting similar findings have been reported in a study on leopard geckos, where a deeper sedation was induced after forelimb compared to hind limb injection of a combination of dexmedetomidine and ketamine [23].

### Monitoring depth of sedation and general anaesthesia, and vital parameters

Monitoring sedation and general anaesthesia are a crucial aspect of ensuring safety. In this systematic review, various reflexes used in studies of crocodiles were evaluated. While the righting reflex was commonly used [1, 3, 4, 6–8, 13, 14], its reliability has been questioned [13]. Other monitored reflexes included the PWR, palpebral reflex, corneal reflex, and position of the palatal valve. Assessment of reflexes, together with vital parameters such as HR, RR, and blood pressure, were used in the studies to monitor the depth of anaesthesia. It is however observed that dissociative drugs such as ketamine can have limited effects on reflex dampening; consequently, maintaining reflexes to some degree during ketamine anaesthesia in combination with low doses of other sedative/anaesthetic drugs should not cause immediate concern [19].

The ten included publications reported varying effects on vital parameters during sedation and general anaesthesia. Cardiac and respiratory depression were a common finding during crocodilian sedation and general anaesthesia especially when medetomidine was used. The included publications using medetomidine claimed that it appeared not life-threatening and as long as medetomidine was reversed with atipamezole no severe postanaesthetic complications were reported [6]. Interestingly it has been speculated [18] that the cardiovascular depression during general anaesthesia was due to the return of HR to normal levels after stress-induced tachycardia associated with capture and handling [18], indicating that the bradycardia observed might not be a complication. The protocol with the least changes in vital parameters, included in the present review, was conducted in American alligators using tiletamine-zolazepam [3]. The procedure was performed at a temperature (28 °C) close to POTZ and all animals were still able to return to sternal recumbency and displayed paddling and head pressing. It could be speculated that the limited impact on vital parameters in the study might be associated with the fact that no alpha-2-adrenoceptor agonist was included in the protocol, but temperature and depth of anaesthesia might have had an impact as well. Possibly more pronounced changes in vital parameters might have been present at lower temperatures and more deep anaesthesia.

Ambient temperature or body temperature were monitored in all ten included publications. Crocodilians are poikilothermic animals exhibiting heart rate hysteresis, a mechanism regulating their HR and allowing them to stay in the POTZ for extended periods during the day and night [24]. This means that HR in crocodilians depends on ambient and body temperature, with HR decreasing as temperature decreases [24]. Temperatures below POTZ were found to make immobilisation less reliable and repeatable, especially when using alfaxalone [8]. A study [7] reported that decreased temperature also results in increased induction time, increased anaesthesia time and decreased number of immobilised animals when using medetomidine; an explanation for this might be the slower HR caused by HR hysteresis. In another study [12] it was speculated that since the regulation of HR hysteresis mainly relies on central autonomic mechanisms, medetomidine, and other drugs might have different effects on HR and blood pressure than we see in mammals.

In three of the included studies animals exhibited bradypnea or apnoea during general anaesthesia [3, 8, 14]. Low venous blood oxygen saturation measurements (36–72% (SvO2)) compared to mammals have been measured in some reptiles [25] and some are able to handle longer periods without breathing during diving [26]. However, normal physiology can change during chemical immobilization, and to the authors’ knowledge, there is no reason not to provide supplemental oxygen via intubation and mechanical ventilation or mask to crocodilians during chemical immobilization.

### Pharmaceutical considerations

The use of NMBAs has been associated with occasional deaths in crocodilians [15] and they have been associated with welfare problems in that they provide neither tranquillizing, analgetic, sedative nor anaesthetic effects [3]. The present review also showed that NMBAs could cause complications, unpredictable recovery times, and have high variability between species [3, 4]. Despite these observations, NMBAs are still being used today [15]. In a case study, pancuronium (a nondepolarizing NMBA) was administered prior to examination of the oesophagus and orotracheal intubation to ensure complete immobilisation and safety of the personnel [15]. After intubation the crocodile was anaesthetised with isoflurane, addressing the possible welfare problems of using NMBAs alone. This illustrates that NMBAs are still used in order to provide enhanced safety for the handler. Animal welfare and appropriate use of NMBAs have been discussed since inadequate anaesthesia can be masked by NMBAs due to limited ability to evaluate depth of anaesthesia and analgesia [27]. If NMBAs are used for crocodilians, it is however important to consider species of crocodilians, doses, and specific agents prior to administration since different species react very differently to NMBAs, and long and unpredictable recovery times have been observed [2, 3].

One of the included studies evaluated the immobilizing effect of IM administration of tiletamine and zolazepam and found the combination useful for capture, transport and minor medical procedures in young American alligators [3]. However, the actual dose reported in the study (15 mg/kg) can be questioned due to missing information on how the drugs were dissolved. The study was published in 1993, and at that time it was recommended to dilute 500 mg of the used product Telazol (250 mg tiletamine and 250 mg zolazepam) with 5 mL sterile water [28]. The final concentration of the solution used for injection was reported to be 100 mg/mL. It is unclear from the study in question whether the displacement effect of the powder was taken into consideration when the concentration in the injected solution was calculated. However clinical relevance of a possible deviation in final concentration due to a displacement effect is expected to be minor since tiletamine-zolazepam is reported to have a high therapeutic index in most species and thereby is considered safe to use [28].

Alfaxalone may be an optimal induction agent for a wide variety of wildlife and exotic pet species, with minimal effects on the cardiovascular system and the possibility for IM administration [29–31]. In different species, complications during recovery are however reported in the form of paddling, minor muscle twitches, or violent motions if alfaxalone is used as the sole anaesthetic agent [30]. A study in crocodilians [8] supported these findings, illustrating unreliable immobilisation with alfaxalone IV. In contrast, a different study, researching the effect of alfaxalone on the green iguana, found that alfaxalone provided reliable immobilisation with no severe recovery complications [29]. However, this study used a much higher dosage of 10–30 mg/kg and administered alfaxalone IM.

A study not present in the selected databases used for present systematic literature search compared ketamine-dexmedetomidine-midazolam (KDM) with alfaxalone-dexmedetomidine-midazolam (ADM) in alligators in the POTZ [30]. The study reported that alfaxalone consistently produced apnoea and intubation was often required whereas the animals maintained spontaneous respiration when immobilised with the ketamine combination. The alligators on ADM did however consistently lose PWR, this was not found using KDM, possibly indicating a deeper anaesthesia and/or analgesia using ADM compared to KDM [31]. However, as mentioned, it is debatable whether the maintenance of reflexes is equal to unsuccessful anaesthesia when using dissociative anaesthetics such as ketamine. The above mentioned complications associated with use of alfaxalone IV in crocodilians [8] were not observed in another study [31] when using alfaxalone in combination with other anaesthetic agents, supporting the theory that most of the recovery complications are only observed when using alfaxalone on its own [30].

An initial dose-dependent depression of respiration is to be expected when administering alfaxalone [8, 29–31]. This effect on crocodilians seems to be increased compared to other reptilian species and intubation and mechanical ventilation are generally provided [8, 30, 31]. As discussed earlier, the effect of prolonged apnoea-induced hypoxia on a semiaquatic reptile is debatable [26].

To provide assisted pulmonary ventilation and allow the administration of supplemental oxygen and/or inhalation anaesthetics, orotracheal intubation in crocodilians is possible after displacement of the gular fold [2, 8, 12, 13, 31]. Three publications included in this review rely on inhalation anaesthesia to enable prolonged anaesthesia times and a surgical level of anaesthesia [9, 12, 13]. Of these, two used isoflurane and one used sevoflurane delivered in a circuit system, all with successful general anaesthesia and subsequent recovery with return of all reflexes.

To allow safe intubation, two studies in Nile crocodiles used a premedication and induction protocol of medetomidine-propofol and medetomidine-ketamine respectively [12, 13]. The first study used low-dose medetomidine only to prolong general anaesthesia after propofol induction, enhance perioperative analgesia, and minimise the risk of arousal during transport [12]. The other study [13] used a premedication protocol very similar to a previous published immobilisation protocol [14]. Medetomidine and ketamine seemed to be an efficient combination for induction to inhalation anaesthesia or translocation and quick procedures such as sex determination, chipping, or other slightly painful procedures [13, 14].

Intramuscular medetomidine (as well as dexmedetomidine) alone seems to be efficient in immobilising crocodilians if administered at sufficient dosages according to surface area, age and body weight and injected in the cranial part of the body at thermal zones above 21 ℃ [6, 7, 15].

Alpha-2-adrenoceptor agonists are reversible with an alpha-2-adrenoceptor antagonist such as atipamezole and are thus often used together. Several of the included publications using medetomidine as an immobilising agent used atipamezole to reverse the effects of medetomidine [6, 7, 12–15]. A few publications discovered that without atipamezole re-sedation could occur if the animals were left unstimulated after anaesthesia [6, 15]. Other complications include distension of the coelom, bradycardia, and slower responses along with a reluctance to eat and bask [15]. These observations seem to make it advisable to use atipamezole as a reversal after the use of alpha-2-adrenoceptor agonists to avoid complications during recovery [6, 7, 14, 15, 31].

### Limitations

This review has limitations. First, it may not have included all relevant literature due to limitations in the search string used and the databases searched. Language barriers also led to the exclusion of potentially valuable publications. Moreover, the review primarily relies on publications of lower levels of evidence, with only one controlled study included. The assessment of the SOE is subjective, based on factors such as study design, number of animals, blinded assessment of recovery, conflict of interest, and the definition of recovery time. The lack of a standardised method for assessing recovery time across publications lowers the overall SOE. Finally, differences in human habituation between the included studies make the comparison challenging, since wild animals are expected to have a higher stress level during handling compared to human-habituated animals.

## Conclusions

In conclusion, peer-reviewed literature regarding sedation and general anaesthesia of crocodilians was limited, however existing literature reported that sedation and general anaesthesia procedures in crocodilians were influenced by species, age, body weight, temperature, route of administration, and combination of anaesthetic agents.

Different sedative and anaesthetic drugs were used including medetomidine, ketamine, tiletamine, zolazepam, diazepam, propofol, alfaxalone, and/or inhalation anaesthesia. Some protocols included neuromuscular blocking agents as well which can offer practical advantages for immobilization, but must be avoid in un-anaesthetised animals due to welfare and ethical implications. If the purpose of the intervention is non-painful procedures such as sex determination, translocation, or imaging it seems that medetomidine administered IM in the TL provides reversible anaesthesia for a considerable time. Dosages however should be calculated using metabolic scaling, taking into consideration that freshwater crocodiles require higher dosages than saltwater crocodiles, and alligators require lower dosages than saltwater crocodiles. If painful interventions such as wound management, dental care, or surgery are needed, medetomidine seems to be efficient when combined with ketamine providing balanced anaesthesia. One study showed that a combination of ketamine and midazolam with alfaxalone IV could provide a surgical plane of anaesthesia. Alternatively, inhalation anaesthesia is a possibility for prolonged anaesthesia.

Overall, continued research and refinement of protocols are essential to enhance evidence of optimal sedation and general anaesthesia and further research is needed to evaluate the efficacy and safety of different dosages and combinations of sedative and anaesthetic agents in species of the Crocodilian.

## Data Availability

The datasets used and/or analysed during the current study are available from the corresponding author on reasonable request. All data and materials are based on data available in the included publications.

## Abbreviations

ADM: Alfaxalone-dexmedetomidine-midazolam
HL: Hind limb
HR: Heart rate
IM: Intramuscular
KDM: Ketamine-dexmedetomidine-midazolam
N/A: Not applicable
NMBA: Neuromuscular blocking agents
OS: Occipital sinus
PCS: Prospective control study
PICO: Population, intervention, comparison, outcome
POTZ: Preferred optimal thermal zone.
PRISMA: Preferred reporting items for systematic reviews and meta-analyses
PRS: Prospective randomised study
PRSX: Prospective randomised study with crossover
PS: Prospective study
PSX: Prospective study with crossover
PWR: Pinch withdrawal reflex
RPS: Renal portal system
RR: Respiratory rate
SOE: Strength of evidence
TL: Thoracic limb

## References

[1] Olsson A, Phalen D. Comparison of biochemical stress indicators in juvenile captive estuarine crocodiles (*Crocodylus porosus*) following physical restraint or chemical restraint by midazolam injection. J Wildl Dis. 2013;49:560–7. 23778605 10.7589/2012-06-160

[2] Fleming GJ. Crocodilian anesthesia. Vet Clin North Am Exot Anim Pract. 2001;4:119–45. 11217457 10.1016/s1094-9194(17)30054-3

[3] Clyde VL, Cardeilhac PT, Jacobson ER. Chemical restraint of American alligators (*Alligator-mississippiensis*) with atracurium or tiletamine-zolazepam. J Zoo Wildl Med. 1994;25:525–30.

[4] Spiegel RA, Lane TJ, Larsen RE, Cardeilhac PT. Diazepam and succinylcholine chloride for restraint of the American alligator. J Am Vet Med Assoc. 1984;185:1335–6. 6511577

[5] Axelsson M, Franklin CE, Löfman CO, Nilsson S, Grigg GC. Dynamic anatomical study of cardiac shunting in crocodiles using high-resolution angioscopy. J Exp Biol. 1996;199:359–65. 9317958 10.1242/jeb.199.2.359

[6] Olsson A, Phalen D. Preliminary studies of chemical immobilization of captive juvenile estuarine (*Crocodylus porosus*) and Australian freshwater (*C. johnstoni*) crocodiles with medetomidine and reversal with atipamezole. Vet Anaesth Analg. 2012;39:345–56. 22642399 10.1111/j.1467-2995.2012.00721.x

[7] Olsson A, Phalen D. The effects of decreased body temperature on the onset, duration and action of medetomidine and its antagonist atipamezole in juvenile farmed estuarine crocodiles (*Crocodylus porosus*). Vet Anaesth Analg. 2013;40:272–9. 23433120 10.1111/vaa.12008

[8] Olsson A, Phalen D, Dart C. Preliminary studies of alfaxalone for intravenous immobilization of juvenile captive estuarine crocodiles (*Crocodylus porosus*) and Australian freshwater crocodiles (*Crocodylus johnstoni*) at optimal and selected sub-optimal thermal zones. Vet Anaesth Analg. 2013;40:494–502. 23461432 10.1111/vaa.12031

[9] Gatson BJ, Goe A, Granone TD, Wellehan JFX. Intramuscular epinephrine results in reduced anesthetic recovery time in American alligators (*Alligator mississippiensis*) undergoing isoflurane anesthesia. J Zoo Wildl Med. 2017;48:55–61. 28363062 10.1638/2015-0293.1

[10] Page MJ, McKenzie JE, Bossuyt PM, Boutron I, Hoffmann TC, Mulrow CD, et al. The PRISMA 2020 statement: an updated guideline for reporting systematic reviews. BMJ. 2021;372:n71. 33782057 10.1136/bmj.n71 PMC8005924

[11] Richardson WS, Wilson MC, Nishikawa J, Hayward RS. The well-built clinical question: a key to evidence-based decisions. ACP J Club. 1995;123:A12–3. 7582737 10.7326/ACPJC-1995-123-3-A12

[12] Stegmann GF, Williams CJA, Franklin C, Wang T, Axelsson M. Long-term surgical anaesthesia with isoflurane in human habituated Nile crocodiles. J S Afr Vet Assoc. 2017;88:6. 10.4102/jsava.v88i0.1451 PMC6138134 28281769

[13] Monticelli P, Ronaldson HL, Hutchinson JR, Cuff AR, d’Ovidio D, Adami C. Medetomidine–ketamine–sevoflurane anaesthesia in juvenile Nile crocodiles (*Crocodylus niloticus*) undergoing experimental surgery. Vet Anaesth Analg. 2019;46:84–9. 30528216 10.1016/j.vaa.2018.09.004

[14] Heaton-Jones TG, Ko JCH, Heaton-Jones DL. Evaluation of medetomidine-ketamine anesthesia with atipamezole reversal in American alligators (*Alligator mississippiensis*). J Zoo Wildl Med. 2002;33:36–44. 12216791 10.1638/1042-7260(2002)033[0036:EOMKAW]2.0.CO;2

[15] Olsson A, Phalen D. Medetomidine immobilisation and atipamezole reversal in large estuarine crocodiles (*Crocodylus porosus*) using metabolically scaled dosages. Aust Vet J. 2012;90:240–4. 22632288 10.1111/j.1751-0813.2012.00907.x

[16] Hicks JW. The physiological and evolutionary significance of cardiovascular shunting patterns in reptiles. Am J Physiol. 2002;17:241–5. 10.1152/nips.01397.2002 12433978

[17] Holz P, Barker IK, Burger JP, Crawshaw GJ, Conlon PD. The effect of the renal portal system on pharmacokinetic parameters in the red-eared slider (*Trachemys scripta elegans*). J Zoo Wildl Med. 1997;28:386–93. 9523631

[18] Bertelsen MF, Mosley C, Crawshaw GJ, Dyson D, Smith DA. Inhalation anesthesia in Dumeril’s monitor (*Varanus dumerili*) with isoflurane, sevoflurane, and nitrous oxide: effects of inspired gases on induction and recovery. J Zoo Wildl Med. 2005;36:62–8. 17315458 10.1638/04-033

[19] Mosley CAE. Anesthesia and analgesia in reptiles. Semin Avian Exotic Pet Med. 2005;14(4):243–62.

[20] Benson KG, Forrest L. Characterization of the renal portal system of the common green iguana (*Iguana iguana*) by digital subtraction imaging. J Zoo Wildl Med. 1999;30:235–41. 10484138

[21] Schwartz MA, Koechlin BA, Postma E, Palmer S, Krol G. Metabolism of diazepam in rat, dog, and man. J Pharmacol Exp Ther. 1965;149:423–35. 5848053

[22] Bennett RA. A review of anesthesia and chemical restraint in reptiles. J Zoo Wildl Med. 1991;22:282–303.

[23] Fink DM, Doss GA, Sladky KK, Mans C. Effect of injection site on dexmedetomidine-ketamine induced sedation in leopard geckos (*Eublepharis macularius*). J Am Vet Med Assoc. 2018;253:1146–50. 30311528 10.2460/javma.253.9.1146

[24] Seebacher F, Franklin CE. Physiological mechanisms of thermoregulation in reptiles: a review. J Comp Physiol B. 2005;175:533–41. 16047177 10.1007/s00360-005-0007-1

[25] Lewbart GA, Hirschfeld M, Denkinger J, Vasco K, Guevara N, García J, et al. Blood gases, biochemistry, and hematology of Galapagos green turtles (*Chelonia mydas*). PLoS ONE. 2014;9:e96487. 24824065 10.1371/journal.pone.0096487 PMC4019482

[26] Bickler PE, Buck LT. Hypoxia tolerance in reptiles, amphibians, and fishes: life with variable oxygen availability. Annu Rev Physiol. 2007;69:145–70. 17037980 10.1146/annurev.physiol.69.031905.162529

[27] Drummond JC, Todd MM, Saidman LJ. Use of neuromuscular blocking drugs in scientific investigations involving animal subjects. The benefit of the doubt goes to the animal. Anesthesiology. 1996;85:697–9. 8873538 10.1097/00000542-199610000-00001

[28] Lin HC, Thurmon JC, Benson GJ, Tranquilli WJ. Telazol-a review of its pharmacology and use in veterinary medicine. J Vet Pharmacol Ther. 1993;16:383–418. 8126757 10.1111/j.1365-2885.1993.tb00206.x

[29] Bertelsen MF, Sauer CD. Alfaxalone anaesthesia in the green iguana (*Iguana iguana*). Vet Anaesth Analg. 2011;38:461–6. 21831051 10.1111/j.1467-2995.2011.00640.x

[30] Jones KL. Therapeutic review: alfaxalone. J Exot Pet Med. 2012;21:347–53.

[31] Aymen J, Queiroz-Williams P, Hampton CCE, Cremer J, Liu CC, Nevarez JG. Comparison of ketamine–dexmedetomidine–midazolam versus alfaxalone–dexmedetomidine–midazolam administered intravenously to American alligators (*Alligator mississippiensis*). J Herpetol Med Surg. 2021;31:132–40.

